# Therapeutic Drug Monitoring of Antidepressants: An Underused but Potentially Valuable Tool in Primary Care

**DOI:** 10.3389/fpsyt.2022.867840

**Published:** 2022-03-29

**Authors:** Daria Piacentino, Esperia Bianchi, Domenico De Donatis, Vincenzo Florio, Andreas Conca

**Affiliations:** Psychiatric Service of the Health District of Bozen, Bozen-Bolzano, Italy

**Keywords:** psychopharmacology, therapeutic drug monitoring, depression, antidepressants, primary care

## Abstract

Depressive disorders are among the most burdensome diseases globally in terms of prevalence, as well as in terms of quality of life, morbidity, and mortality. Hence, it is becoming increasingly common for primary care physicians to administer and monitor the treatment of individuals affected by depressive disorders. In this framework, Therapeutic Drug Monitoring (TDM) comes to the forefront. TDM is the measurement of specific drugs in the blood or plasma/serum, and its usefulness lies in the fact that it allows physicians to assess drug levels to personalize and optimize treatments. TDM has been used for decades to measure several classes of psychotropic drugs, such as antiepileptics and antipsychotics, but the use of this tool is still in its infancy in regard to antidepressants. In the context of primary care, TDM of antidepressant drug treatment shows promise, as it can enable primary care physicians to monitor the safety and efficacy of the treatment, leaving to secondary care, i.e., psychiatrists, the management of the more complex clinical cases.

## Introduction

Depressive disorders are highly prevalent worldwide, involving individuals of all ages and significantly impairing the quality of life of the affected individuals. A 2020 systematic review reported the lifetime prevalence of major depressive disorder (MDD) to range worldwide from 2 to 21%, reaching the highest rate in Europe (1). Similar numbers were observed by a 2018 study on the prevalence of MDD in 36,309 US adults, which found that the 12-month and lifetime prevalence of DSM-5 MDD was 10.4 and 20.6%, respectively (2). In addition to the high incidence and prevalence of MDD, it is also important to underline the cumbersome burden of this disease on several aspects of life, such as morbidity and mortality (3, 4), employment, familial stability, and education (5). Remarkably, in 2017, the World Health Organization (WHO) assessment of the global burden of disease, showed depression to be among the top three leading causes of years of life lost due to disability (6).

## Depressive Disorders in Primary Care

Ideally, all patients suffering from depressive symptoms should be treated via a multidisciplinary approach by their primary care physician together with a psychiatrist (7). However, mainly due to the stigma still associated with the consultation of a psychiatrist, it is estimated that up to 60% of mental health care delivery takes place in the setting of primary care services (8). A 2009 study by Mark and colleagues showed that, in the U.S., 62% of antidepressant prescriptions are given by primary care physicians (9). Indeed, primary care physicians are those in direct contact with individuals affected by MDD and other depressive disorders and play a key role not only in screening these disorders, but also in diagnosing and treating them, with the latter aspect including therapeutic monitoring. As concerns screening, the U.S. Preventive Services Task Force (USPSTF) has recently recommended that all adults, regardless of risk factors, should be screened for depression in primary care settings, given that early recognition and prompt treatment are known to decrease morbidity (10). Following diagnosis and initiation of treatment, the role of the primary care physician is to monitor the patient's clinical status and its possible changes. Nowadays, this process mainly occurs via an interview taking place at a primary care facility (11, 12). Closely related to the role of the primary care physician in monitoring the course of the disorder, is the monitoring of both treatment effectiveness and compliance. It is estimated that, in the U.S., rates of non-compliance to antidepressant drug treatment among older adults range from 29 to 40% (13). For all these reasons, it is important for primary care physicians to be able to optimize antidepressant drug treatment, as they often find themselves administering these drugs and adjusting drug dose.

## What is Therapeutic Drug Monitoring (TDM)?

Therapeutic drug monitoring (TDM) is the measurement of a drug in the blood or plasma/serum of the patient, taken at regular intervals to optimally tailor the treatment to such patient, thus keeping the blood levels of the drug within a given therapeutical window (14). In research, TDM has been used since the 1960s, and its first foray into clinical practice took place in the 1970s, when TDM was used to monitor the administration of those drugs that are known to have a very narrow therapeutic window, i.e., their minimal effective concentration is very close to their minimal toxic dose. Drugs in which TDM played, and continue to play, a crucial role are, among others, digoxin (15), phenytoin, and lithium, for which the use of TDM is widespread (16). Currently, however, the usefulness of TDM is not limited to preventing drug toxicity while maintaining efficacy, but it includes other aspects of pharmacological monitoring in clinical practice, including a better understanding of the possible drug-drug interactions in patients with polytherapy (17), as well as monitoring treatment compliance (18).

Despite TDM being a useful tool available to physicians, it has some limitations. First, the timing of drug measurement must be taken into account. There are usually two acceptable timings of measurement, i.e., the peak concentration time (or zenith), which presents major intra- and inter-individual variations in the rate of absorption, and the trough concentration time (or nadir), which precedes closely the following dose (19). This makes it more complex to plan and carry out the sampling in an outpatient setting. A second issue that could play a role in the attainability of TDM in a wider clinical setting outside of research is the cost of the procedure. Assays such as ELISA, which is commonly used to measure TDM, are not inexpensive, and these costs must be considered and weighted against the clinical benefits that would be obtained by a more widespread use of TDM (20). Last, despite the usefulness of TDM, not all drugs warrant its use in clinical practice. A 1991 study by Aronson and colleagues (21) describes the main criteria that make a drug a good candidate for TDM, and these criteria are still valid nowadays:

1) difficulty in interpreting clinical evidence of therapeutic or toxic effects;

2) a good relationship between plasma drug concentration and therapeutic or toxic effect, or both;

3) a low toxic to therapeutic ratio;

4) dose does not metabolize to important active metabolites.

So far, there are several classes of drugs that are recognized as fulfilling these criteria, such as antiepileptics (22), some antibiotics and antimycotics (23), antiretrovirals (24), immunosuppressants (25), antipsychotics (26, 27) and, more recently, antidepressants (28, 29).

## TDM of Antidepressants Drug Treatment in Primary Care

Antidepressant drug treatment aims to induce remission without causing adverse effects during the acute phase of the disorder, and prevent relapse or recurrence during continuation or maintenance therapy. To reach these goals, drug choice and dose must be optimized for each individual patient. TDM of antidepressant drug treatment, which assumes that clinical effects correlate better with drug blood levels than drug doses, can be helpful. Specifically, it can be helpful in primary care settings for:

1) *Compliance monitoring*. Due to the long course and chronic nature of antidepressant drug treatment, self-discontinuation or non-regular intake of the drug is common (30). This is partly attributable to the delayed onset of action of antidepressants, which can take weeks to months to reach their full effects, with patients initially perceiving the treatment as ineffective (31). Non-compliance may lead to suboptimal treatment outcomes. TDM provides an objective means to monitor compliance;

2) *Treatment optimization*. By collecting data regarding both drug dose and drug blood levels, decision-making related to antidepressant drug treatment can be optimized (32). This translates into both the reduction of side effects (33) and the avoidance of subtherapeutic concentrations, which would lead to little or no effectiveness of the drug itself (34);

3) *Toxicity avoidance*. Any antidepressant drug treatment should be safe. Different individuals, despite receiving the same drug dose, can achieve substantially different blood levels of the drug. For those drugs with a small therapeutic window and in presence of comorbid diseases and/or polytherapy, TDM can be useful in identifying those patients at risk of toxicity within “therapeutic doses”(35).

4) *Idiosyncratic reactions documentation*. In patients who experience idiosyncratic drug reactions, i.e., drug reactions that occur rarely and unpredictably, TDM can play a relevant role in measuring the blood levels of the antidepressant drug that caused the reaction and reveal whether the patient reached unusually high levels of the drug for the given dose (36).

The above-mentioned problem of cost-effectiveness of TDM in clinical practice can be even more relevant in primary care settings compared to hospital settings. In fact, the high costs of this tool may prevent its implementation. Unfortunately, this also applies to antidepressants, despite them being, as previously stated, among the most commonly prescribed psychotropic drugs in the general population (37). However, as a consensus of experts on the topic aptly put in 2005: “TDM should be limited to situations where it may be expected that the result will help to solve a therapeutic problem” (38). This argument is particularly valid in the analysis of cost-benefits. It is important to take into account not only the cost of TDM itself, but also how monitoring the drug concentration could help optimize resources, avoiding the extra costs (both time- and in money-wise) related to a prolonged process of dose titration based only on clinical response, as well as the costs of treatment due to concentration-dependent adverse effects (35). Additionally, TDM would allow for fast switching of drug class in non-responders, who, in the context of depressive disorders, make up to 40% of patients (29).

Below we briefly describe seven clinical cases of individuals with MDD who were treated with escitalopram at our hospital's outpatient clinic and monitored with TDM (28).

## A Case Series Showing the Usefulness of TDM of Escitalopram at the Department of Psychiatry of Bozen, Bozen-Bolzano, Italy

Seven outpatients with DSM-5 MDD were evaluated at baseline (t0) and after 4 weeks (t1). These patients had a mean age ± standard deviation of 46.4 ± 18.1 years and 4/7 (57.1%) were women. After validation of MDD diagnosis, these patients were treated with escitalopram. At steady-state, we collected blood samples from these patients and measured plasma concentrations of escitalopram. Escitalopram is the (S)-enantiomer of the racemic selective serotonin reuptake inhibitor (SSRI) antidepressant citalopram. It is an effective and well-tolerated drug used in the treatment of MDD. In the dose range of 10–30 mg/day, it shows linear and dose-proportional pharmacokinetics. The molecule shows a high affinity for the serotonin transporter and binds to an allosteric site that increases the drug's efficacy. After oral administration, steady-state concentrations are achieved within 7–10 days. Despite intra- and inter-individual and overall pharmacokinetic variability, the therapeutic index, according to the TDM group of the Arbeitsgemeinschaft fur Neuropsychopharmakologie und Pharmakopsychiatrie (AGNP), ranges between 15 and 80 ng/ml (26, 39).

**Figure 1:**
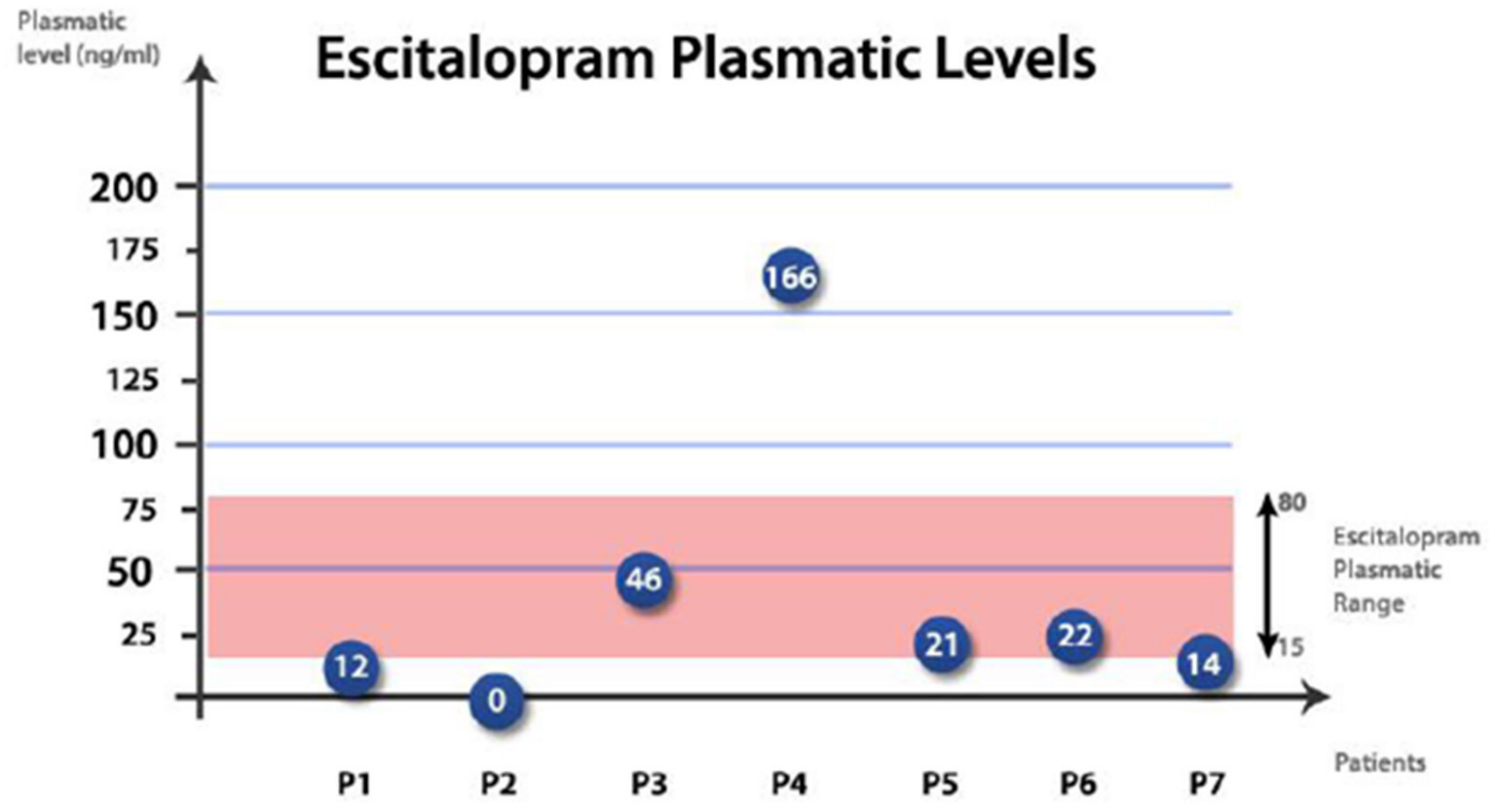
Plasma levels of escitalopram in our seven patients.

As reported in Figure 1, three patients of our sample showed therapeutic plasma levels of escitalopram, i.e., levels within the optimal range (15–80 ng/ml). Patient 1 (female, 38 years old) had lower plasma levels than the therapeutic ones, but we did not modify the dose of escitalopram, since the clinical status was satisfactory. Patient 2 (male, 55 years old) was not compliant with the treatment and stopped taking escitalopram altogether. This explains why the plasma levels of the drug are equal to zero. Patient 3 (male, 57 years old), though having plasma levels of escitalopram within range, initially reported mild side effects (restlessness). The latter disappeared after few days of taking the drug and we did not modify the drug dose. Patient 4 (male, 79 years old) had plasma levels of escitalopram significantly above the therapeutic ones and reported severe side effects (palpitations, restlessness, and drowsiness), thus we rapidly decreased the drug dose. Patient 5 (female, 46 years old) had therapeutic plasma levels of escitalopram, showed a stable clinical status, and did not report side effects. The same applied to patient 6 (female, 30 years old). Patient 7 (female, 20 years old) had slightly lower plasma levels than the therapeutic ones and her clinical status was unsatisfactory, thus, in agreement with her, we increased the dose of escitalopram. In all seven patients, it was possible to optimize clinical status by simultaneously considering the plasma concentrations of escitalopram and the clinical data. In five of the seven patients, the dose of escitalopram was modified based on TDM, by monitoring the plasma concentrations of the drug.

## Conclusions

TDM of antidepressant drug treatment is an underused tool, given its ability to optimize treatment of individuals affected by major depressive disorder or other depressive disorders, as well as its usefulness to monitor and potentially improve treatment effectiveness and compliance. This is especially true in primary care, where prescription, monitoring, and adjustment of antidepressant drug treatment often take place. It is our belief that the use of a laboratory tool such as TDM, together with the assessment of the overall clinical status of the patient being administered the antidepressant drug treatment, would facilitate decision-making in primary care settings.

## Author Contributions

All authors listed have made a substantial, direct, and intellectual contribution to the work and approved it for publication.

## Conflict of Interest

The authors declare that the research was conducted in the absence of any commercial or financial relationships that could be construed as a potential conflict of interest.

## Publisher's Note

All claims expressed in this article are solely those of the authors and do not necessarily represent those of their affiliated organizations, or those of the publisher, the editors and the reviewers. Any product that may be evaluated in this article, or claim that may be made by its manufacturer, is not guaranteed or endorsed by the publisher.
